# The transition of cannabis into the mainstream of Australian healthcare: framings in professional medical publications

**DOI:** 10.1186/s42238-021-00105-w

**Published:** 2021-11-21

**Authors:** Monique Lewis, John Flood

**Affiliations:** Griffith University, Queensland, Australia

**Keywords:** Medicinal cannabis, Content analysis, Healthcare, Doctors, Media, Framing

## Abstract

**Background:**

Medicinal cannabis has been legalised for use for a range of specified medical conditions in Australia since 2016. However, the nature of the government regulations and the subsequent complexity of prescribing, as well as doctors’ safety uncertainties and the stigma of the plant, remain contributing barriers to patient access. Media representations can offer insights into the nature of the discourse about new medical products and therapies and how ideas and understandings about social phenomena become constructed. Focusing on professional medical publications, this study sought to investigate how medicinal cannabis is being represented in professional medical publications.

**Methods:**

Using a content analysis approach, we investigated articles about medicinal cannabis from 2000 to the end of 2019 in the *Medical Journal of Australia, Australian Doctor*, *Medical Observer*, *Australian Journal of General Practice*, *Australian Family Physician*, and *Australian Medicine.* Articles were coded according to article type, framings of cannabis, headline and article tone, and key sources used in the article. We also used manifest textual analysis to search for word frequencies, and specific conditions referred to in the articles retrieved.

**Results:**

A total of 117 articles were retrieved for analysis, the majority of which were news stories for a physician audience. Across the longitudinal period, we found that most reports carried a positive tone towards medicinal cannabis. Cannabis is most frequently framed as a legitimate therapeutic option that is complex to prescribe and access, does not have a strong evidence base to support its use, and also carries safety concerns. At the same time, the outlook on cannabis research data is largely positive. Primary sources most frequently used in these reports are peer-reviewed journals or government reports, voices from medical associations or foundations, as well as government and university researchers. Chronic pain or pain were the conditions most frequently mentioned in articles about cannabis, followed by epilepsy, cancer or cancer pain, and nausea and chemotherapy.

**Conclusions:**

This analysis offers evidence that medicinal cannabis is being framed as a valid medicine advocated by the community, with potential for addressing a range of conditions despite the lack of evidence, and a medicine that is not free of risk.

## Background

This article analyses media representations of medicinal cannabis in Australian medical publications. Legalisation occurred in Australia in 2016. Firstly, we contextualise cannabis in terms of its historical usage, its legal status, and research relating to knowledge, attitudes, and usage. Secondly, the media framing of medicinal cannabis is introduced in connection with Australian medical journals. As sources of medical information, they are relatively under-researched. Thirdly, our methods are outlined followed by the results of the framings, which show a gradual opening up to and receptivity by the medical profession towards medicinal cannabis. Finally, we discuss the findings and conclude that medicinal cannabis is on a journey towards becoming a legitimised medicine.

The Cannabis sativa plant has an incredibly multi-layered, rich, and versatile history of human uses for food and fibre, as well as recreational, and religious and spiritual purposes throughout the world (McPartland and Hegman 2018; Aldrich 1997; Touw 1981; Li 1974; Bonini et al. 2018; Frankhauser 2008). It also has an extensive and diverse history of medicinal use. The cannabis plant is most likely to have originated in the north-eastern Tibetan Plateau, with archaeological evidence tracing its multiple range of uses back to Paleolithic and Neolithic times (McPartland and Hegman 2018; Touw 1981). Its use in China, Tibet, India, Nepal, Japan (McPartland and Hegman 2018, Touw 1981), Ancient Greece and Rome (Butrica 2002), and Iran, Iraq, Turkey, Syria, and the Balkans (McPartland and Hegman 2018; Lozano 1997; Aldrich 1997) goes back thousands of years. Bonini et al. (2018) note its continued medicinal use amongst indigenous societies in regions of North Pakistan, Nepal, Uganda, Kenya, and the Caucasus.

Since ancient times, the use of medicinal cannabis has been documented for a wide range of ailments in regions like India, China, Tibet, and Mesopotamia, for conditions such as epilepsy, dysmenorrhea and labour pain, rheumatism, urinary tract infections, gonorrhoea, and even leprosy. It was also used as a topical treatment for haemorrhoids, ear infection, and wounds (Aldrich 1997; Touw 1981). Its adaptation in Western countries like the UK has been well documented, where it was used in the nineteenth and early twentieth centuries as an analgesic, anti-spasmodic, appetite stimulant, and topical anaesthetic (Aldrich 1997).

The recreational use of cannabis is well known and attributed to its power as a psychotropic plant, specifically due to the cannabinoid tetrahydrocannabinol (THC). Across the twentieth and twenty-first centuries, this has been a primary association and stigma for the plant, to such an extent that that its many other uses were erased from the memories and pharmacopoeias of those Western, industrialised countries that effectively banished it from holding any legitimate place in society, including as a medicine (Newhart and Dolphin 2019; Ferraiolo 2007). In addition to Western anxieties regarding its psychotropic capacities and its use by often stigmatised ‘deviant’ citizens as an intoxicating substance, cannabis also competed with the whole armoury of newer and stronger ‘heroic’ medicines of the early twentieth century (Lewis and Flood 2019). These new and standardised preparations—such as the isolation of opioids from the opium poppy to make opiate medicines like morphine, as well as the analgesic application of newly discovered aspirin (Henry et al. 2016; Sznitman et al. 2008)—were difficult for any plant to compete with, especially one whose active constituents would not be revealed for another 30 years (Atakan 2012).

The discovery of the endocannabinoid system shed light on how plant-derived cannabinoids like cannabidiol (CBD) and tetrahydrocannabinol (THC) interacted with human and animal systems. Research into the plant’s chemical compounds has also revealed a rich array of terpenes and flavonoids that may also account for the plant’s therapeutic effects (Kotsirilos and McGregor 2021). Despite there being relatively little we know about the effects of cannabis, there is increasing research on its medical effects with varying degrees of success and effectiveness. Bostwick (2012:174) notes that medical and recreational uses in the USA have now blended together and are becoming indistinguishable in the eyes of citizens. Within the USA, 37 states have legalised the use of cannabis for medicinal and/or recreational uses even though the federal government upholds its illegality (NCSL 2021). Elsewhere, cannabis has become legal completely in the case of Uruguay and Canada, and partially in other countries.

Before 2016, Australian law considered cannabis an illegal drug. In February 2016, the Narcotics Drug Amendment Act 2016 established a national licensing and permit scheme for the cultivation, production and manufacture of cannabis for medicinal and scientific research purposes. The Office of Drug Control (ODC) (ODC 2020), alongside the Therapeutic Goods Administration (TGA), regulates the production of cannabis and cannabis products. Patients can legally access medicinal cannabis through a doctor, via the TGA’s Authorised Prescribers and Special Access schemes.

The TGA (2018) has approved the use of medicinal cannabis for:

- Chemotherapy-induced nausea and vomiting
- Refractory paediatric epilepsy
- Palliative care indications
- Cancer pain
- Neuropathic pain
- Spasticity from neurological conditions
- Anorexia and wasting associated with chronic illness (such as cancer)

The TGA regulations permit applications for most cannabis products but nevertheless distinguish between cannabis products based on CBD and THC. The latter remains a dangerous drug and is considered higher risk for treatment than CBD (TGA 2019). Australian health authorities advise that cannabis should not be used as a first line of treatment (Kotsirilos and McGregor 2021).

In 2018, the Federal Minister for Health announced that the government wanted Australia to become the largest exporter of medicinal cannabis in the world (Guardian 2018). This was followed by a TGA report in 2019 reviewing the 2016 amendments. McMillan's “Review of the Narcotic Drugs Act 1967” (McMillan 2019:1-4) made 26 recommendations which the Health Minister accepted. The review, however, did not cover patient access to medicinal cannabis. Licensing was recommended to be simplified—one licence instead of three—and the period to be extended from 3 to 5 years. The states’ situation varies substantially. Victoria is in the vanguard, legalising cannabis for medicinal use in 2016. NSW is investing heavily in the medicinal cannabis industry and eased patient access in 2018. The remaining states are playing catch-up with Victoria and NSW but all seem to pulling in the same direction and see both the economic and medicinal benefits of cannabis. The outlier is ACT which in 2020 legalised cannabis for recreational use. While it may be legal in ACT it remains illegal under Commonwealth law and the legal situation remains fluid (Senate Community Affairs References Committee 2020).

A recent study by Lintzeris et al. (2020) measured attitudes and usage of medical cannabis amongst 1388 Australians, in the wake of changes in legislation permitting (restricted) medical access. The majority of respondents to the ‘CAMS-18’ online survey believed that they should be able to bypass doctors’ approval for access to cannabis, that it should be part of routine healthcare in Australia and that its costs should be subsidised by the government (Lintzeris et al. 2020). A high proportion of participants also believed that medicinal cannabis should meet safety standards and thought that the existing regulatory system was inadequate. The high costs of legally accessed cannabis caused concern amongst over half of respondents who also felt the current model for access was difficult for patients to navigate (Lintzeris et al. 2020). Despite its legal availability, most consumers in this study reported accessing cannabis products illegally and were uncertain about the quality or composition of cannabis products (Lintzeris et al. 2020). Although this is not a large survey—given the proportion of Australians currently believed to be using cannabis for healthcare reasons, and is more broadly representative of illicit users rather than those with prescribed access—it does offer insights into some of the main reasons for usage and users’ expectations about access and regulation.

Evaluating the levels of knowledge about cannabis amongst Australian general practitioners (GP) is important because they are gatekeepers and prescribers for legal medicinal cannabis access. A 2018 study of 640 Australian GPs published in the British Medical Journal (Karanges et al. 2018) sheds some light about the knowledge and attitudes of GPs regarding medicinal cannabis. The survey findings showed that GPs rated their knowledge of medicinal cannabis as poor, in terms of how patients can legally access it, how it is regulated, the effects of the medicine, and the products available. The study also showed that doctors were mostly supportive of medicinal cannabis where there was a strong evidence base for it; e.g. palliative care, chronic cancer pain, intractable epilepsy, nausea and vomiting from chemotherapy, and spasticity in multiple sclerosis. The support amongst doctors for usage was low in other conditions such as depression, anxiety, and insomnia, for which gold-standard scientific evidence is either minimal, weak, or negative (Bonaccorso et al. 2019; Sarris et al. 2020). Yet, patient-reported use of medicinal cannabis for pain, anxiety, and depression is high (Kosiba et al. 2019), and six US states have permitted post-traumatic stress disorder as a treatable condition by medicinal cannabis (Bridgeman and Abazia 2017). This study sets the scene quite effectively to help us understand how doctors are considering cannabis as a legitimate therapeutic option in their practice—as well as how it is being reported on in their professional publications.

Understanding medicinal cannabis in the current health and media landscapes is complex. On the one hand, its twentieth century trajectory has carried immense social stigmatisation for users. In the twenty-first century, we are seeing cannabis gaining legitimacy as a mainstream, though precarious, medicinal substance. In Australia, this option is not controlled by lay people however, but depends on a doctors’ willingness to prescribe it. Media representations offer insights into how cannabis is being constructed as a medicine in both public and professional spheres.

### Media framing of cannabis

Mediated representations of medicinal cannabis, surprisingly, have not received much attention in the scholarly literature. Researchers in Israel, Sweden, Estonia, and the USA have investigated media discourses about medicinal cannabis (Sznitman and Lewis 2015; Lewis et al. 2015; Kaiser 2011; Mortensen et al. 2018; Månsson 2016, 2017; Abalo 2019a, 2019b; Paimre 2017). These have been mostly newspaper-based studies, with the exception of Sznitman and Lewis (Sznitman and Lewis 2015; Sznitman and Lewis 2018), which were audience-reception studies based on video and web-based resources developed by the researchers. This literature indicates the extent to which medicinal cannabis is increasingly being represented in the context of legalisation and constructed as a legitimate medical product. This is the first study to investigate how cannabis is being constructed in GPs’ publications, as well as the first study of mediations of medicinal cannabis in Australia.

### The role of medical journals

Our research focuses on discourse about cannabis in several major medical journals and magazines for Australian doctors. Medical journals function as the index of the medical profession’s knowledge and expertise. Medical journals as a phenomenon have not attracted a lot of scholarly attention despite their social, economic, cultural, and political impacts (Bynum et al. 1992). They have existed since 1731, when Britain’s first professional medical journal, *Medical Essays and Observations* was launched (Booth 1982). Since the eighteenth century, medical journals have acted as vehicles for the profession in numerous ways. They restricted information to medical peers and colleagues, keeping ‘specialised’ content from lay audiences (Porter 1992). They enabled professional political activism and dissent (Booth 1982), as well as presenting peer-reviewed case studies, research reports, observations, and opinions for members of the profession. Taking into account neo-Weberian and Marxist perspectives (Saks 2012, 2016; Baer 2010; Freidson 1970; Turner 1995; Willis 1989; Abbott 1988; Starr 1987), professional medical journals also work to secure and reinforce professional closure and dominance, which enhances members’ social and financial capital, not to mention their power over market interests (Bourdieu 1986; Coleman 1988; Puttnam et al. 1993).

In modern times, medical journals influence a wider group of people than their specific targeted readerships (Smith 2006; Entwistle 1995), encompassing health and medical journalists who use them as important expert sources for news stories (Entwistle 1995; Van Trigt et al. 1994). Non-expert citizens also read these news stories or encounter professional journal abstracts and articles during web searches. Politicians, policymakers, and legislators are important audiences who are influenced and, of course, specifically targeted by medical journals (Stryker 2002). Social capital is afforded to the medical profession due to the very exclusivity of these publications that speak specialised, professionalised languages reinforcing the distinctiveness of the profession’s medical expertise and knowledge from the public sphere and everyday discourse. Doctors also rely on professional publications to keep abreast of developments in their profession, such as insurance issues, legal proceedings, case studies, discovery of new medicines, and new research discoveries and breakthroughs.

Although these sources are under-explored (Bynum et al. 1992), representations of medicinal cannabis in them are even more rare. These publications offer valuable indicators for current debates and disputes, political contestations, demands for reform or policy change, reactions to research activities and evidence, as well as attitudes from the medical profession towards new treatments and therapies like medicinal cannabis. We consider reports about cannabis in Australia’s leading medical journal, the *Medical Journal of Australia*, as well as four other publications that have been selected for their high readership and accessibility: *Australian Doctor*, *Medical Observer*, *Australian Journal of General Practice*, and *Australian Medicine*. These publications also focus on GPs, who are the first port of call for patients who want to be prescribed medicinal cannabis. We were interested to investigate how these publications have been framing medicinal cannabis, given its relatively recent arrival on the Australian healthcare scene.

## Methods

### Mapping media frames

Understanding and recognising how stories about a particular phenomenon are framed is a crucial part of media literacy. In order for frames—which offer what Goffman (1974) called a ‘schemata of interpretation’—to resonate with us, these frames must appeal to our existing belief systems, values, narratives, and ideologies, as Lakoff noted (Lakoff 2006). Framing is not always obvious and so tends to be connotative. Authors of texts may use more surreptitious, and sometimes unconscious, literary devices such as rhetoric, trope, metaphor, or juxtaposition—techniques that evoke moral positions, predictions of possible effects, and prescribe solutions (Entman 1993; Johnson-Cartee 2005; Weaver 2007). A latent analytical approach to content analysis enables the identification of main themes, how certain aspects of the story being told are emphasised, and the intonation of the article, which may be positive, negative, neutral, or mixed (Entman et al. 2009). In essence, framing involves consistent construction of facts, and offers opportunities for claims-makers (and ‘truth-claims’) to compete to persuade audiences. Media frames depend on patterns reflecting the organisation and interrelation of ideas, which is why systematic approaches are effective (Kitzinger 2007). By mapping how professional medical publications frame medicinal cannabis, we are able to capture a sense of how the cannabis plant-as-medicine, its by-products and all its possibilities in its mediatised state may be presented to and possibly perceived by the medical professions.

### Data collection

A search was conducted from 2000 to the end of 2019 in the following publications for GPs: *Australian Doctor*, *Medical Observer*, *Australian Journal of General Practice*, *Australian Family Physician*,^1^ and *Australian Medicine*. The online versions of these publications were selected given their popularity as information sources for GPs and other medical professionals. We also included the Australian Medical Association’s (AMA), peer-reviewed scholarly journal, *The Medical Journal of Australia* (MJA). The search terms used were as follows: “medicinal cannabis”; “cannabis”; “marijuana”; “medic* marijuana”; and “medic* cannabis”.

As GPs are the first port of call for patients who may seek medicinal cannabis, we selected GP publications with high readerships and followings, as well as the AMA’s own peer-reviewed scholarly journal. The publications were also chosen for their availability and accessibility. All items referring to medicinal cannabis were included. Articles referring to use or abuse of cannabis as a recreational drug without mentioning medicinal cannabis were excluded from the dataset.

Articles were sourced from the start of 2000 to the end of 2019. The units of analysis for this content analysis were article type (e.g. news piece, case study, letter to editor, editorial), framings of medicinal cannabis, headline and article tone,^2^ and key sources used in the article. Any duplicate articles that appeared within the same publication were deleted. After being briefed on definitions for each coding category and receiving a codebook as a guide, two research assistants independently coded 22 matching articles. The coders then met with the chief investigators to discuss the coding results, gauge the level of agreement between coders, and clarify any areas of difference. One coder then independently coded the entire dataset and entered the data into NVivo v12.

The coding method was a combination of deductive and inductive codes. These categories were based on a priori frames, drawing on earlier media representation research into mainstream news and biomedical representations of herbal medicine and complementary medicine in Australia (Lewis 2011a; 2011b; Lewis 2015; 2019), as well as cannabis-specific research (Lewis, Broitman 2015; Sznitman and Lewis 2015). A number of new frames also emerged during the coding process (e.g. framing of medicinal cannabis as a community-driven phenomenon, or patients self-prescribing and accessing cannabis on the ‘black market’ to deal with health problems). Manifest textual analysis was also undertaken, using NVivo to search for word frequencies, which gave us an indication of the most common words across the articles about cannabis, and the contexts in which they appeared. This offers a more denotative approach to complement the data. We also conducted text searches in NVivo for specific conditions referred to in these articles, such as: pain, chronic pain, epilepsy, and multiple sclerosis.

## Results

A total of 117 articles were retrieved for the analysis. We sourced articles from all publications, with the exception of *Australian Family Physician*, which yielded no results for references to medicinal use of cannabis. The vast majority of articles were news stories for a physician audience (*n* = 81). There were 8 original research or review papers, followed by editorial or opinion pieces (*n* = 14) and general information or guidelines for practitioners (*n* = 6). Three articles were audience polls about medicinal cannabis and three were sponsored content (e.g. upcoming seminars). One letter to the editor was retrieved from the *MJA*. There was one case study and one book review during this period.

**Fig. 1:**
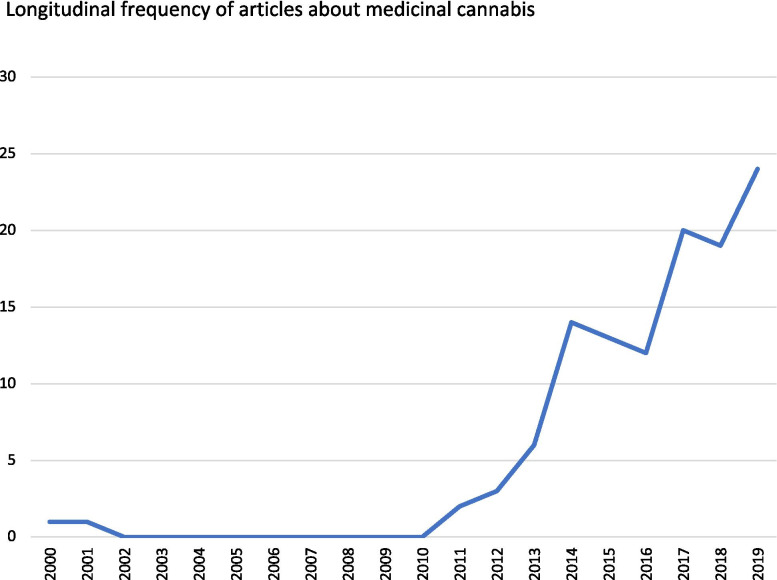
Longitudinal frequency of articles about medicinal cannabis

The number of articles across the longitudinal period can be viewed in Fig. 1. Only one article appeared in the MJA in 2000 and again in 2001. However, from 2010, a dramatic increase is observable in articles about medicinal cannabis in the publications under analysis. After the lull between 2002 and 2010, cannabis articles gather momentum, substantially increasing in 2014 and then gradually rising each year (with the exception of 2017 and 2018 which have the same number), with the highest rates seen between 2017 and 2019.

The most prevalent sources drawn upon in these articles came from: journals or reports (*n* = 34) (of which 25 were peer-reviewed medical journals and 9 were government or medical association reports); spokespeople from medical associations or foundations (*n* = 25) such as the Australian medical Association (AMA) or the Royal Australian College of General Practitioners (RACGP); voices from government (*n* = 18); university researchers (*n* = 14); and medical personalities (*n* = 9). Less frequent sources included doctors who are registered cannabis prescribers (*n* = 6); TGA representatives (*n* = 3), laypeople (*n* = 1); and doctors who are represented as hospital-based practitioners (*n* = 1). Voices from private research and the corporate sector occurred in only one article each across the entire longitudinal period.

**Fig. 2:**
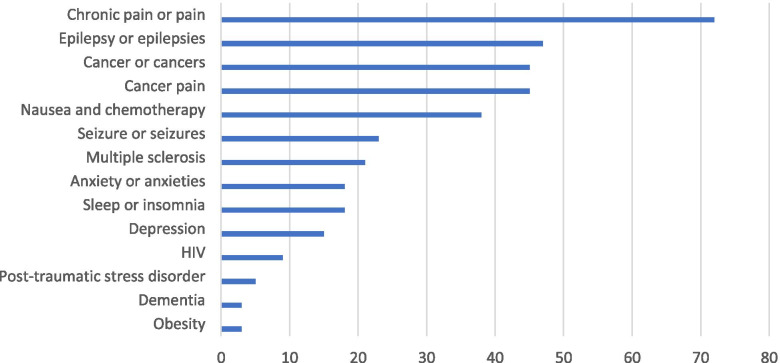
Conditions most frequently mentioned

Figure 2 shows the conditions most frequently referred to across the articles, based on word frequency searches in NVivo. Pain and chronic pain were the most common conditions mentioned over the time period and were referred to in 62% of all articles. Epilepsy or epilepsies were mentioned in 39% of articles. Cancer pain specifically was articulated in 38% of articles followed by nausea and chemotherapy in 33% of articles. Less frequent were references to multiple sclerosis, anxiety and depression, sleep, and HIV.

### Headline and article tone

**Fig. 3:**
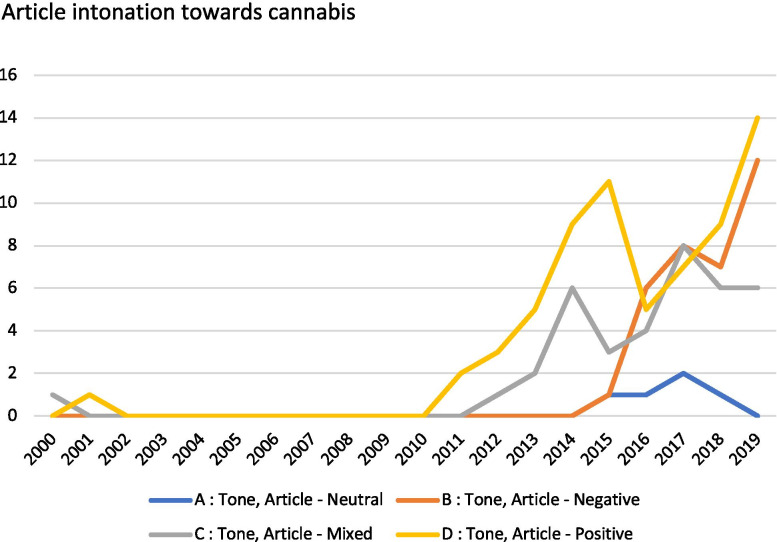
Article intonation towards medicinal cannabis

Overall, more articles carried positive headlines (*n* = 59) than negative ones (*n* = 43), with a lower frequency of neutral media pieces (*n* = 15). Figure 3 charts the results for article tone across the time period. The overall tone of articles was positive towards cannabis (*n* = 55), with a substantially lower rate of mixed (*n* = 29) and negative (*n* = 26) intonation. Only seven articles carried a neutral tone towards medicinal cannabis. No negative articles appear in these medical publications until 2015. In contrast, 2017 is the only year in which negative and mixed articles outweigh positive ones. We do see the highest rate of negative articles in 2019 (*n* = 10), with 10 articles also carrying a positive tone towards cannabis, and five being mixed in tone.

Most positive framings were framings about legitimacy of cannabis, legalisation, and positive research findings. Framings that more often carried a negative tone were related to poor evidence and safety issues. We were interested to gauge whether anecdotal information or narratives were used in relation to medicinal cannabis. There were 11 articles drawing on positive anecdotes about medicinal cannabis, in comparison to two articles from the Australian Doctor containing a negative anecdote during the time period.

### Cross-tabulation of sources

The difference in tone across sources was most apparent with government sources and prescribing practitioners, where positive intonation was much more pronounced. Government voices and medical journals were the most frequent sources used in articles with a positive tone. Medical journals were also the most frequent for negative tone. A relatively even spread of positive, negative, and mixed article tone also occurred with medical association or foundation spokespeople and university researchers. The high rate of reference to reports from medical journals signals the value of peer-reviewed research for professional medical publications.

### Framings

**Fig. 4:**
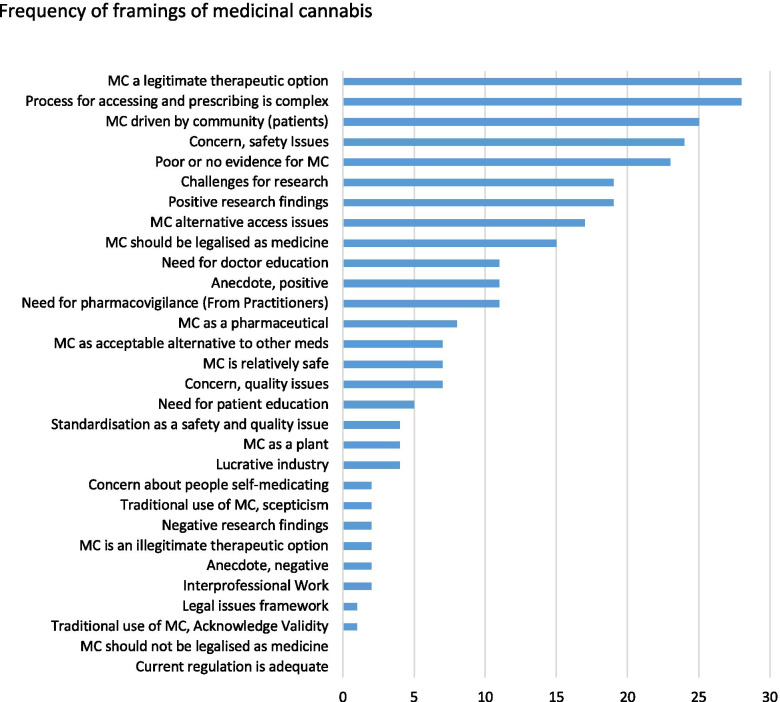
Frequency of framings of medicinal cannabis

The most frequent framings (see Fig. 4) occurring in this study across the time period positioned medicinal cannabis as a legitimate therapeutic option (*n* = 28), framed cannabis as a medicine whose regulation is being driven by the community (*n* = 28), addressed the complexity of prescribing and patient access (*n* = 25) and the lack of evidence (*n* = 24), and conveyed safety concerns (*n* = 23). Cannabis was more frequently portrayed as a pharmaceutical medicine or drug (*n* = 11) rather than a plant product (*n* = 4). Concern about safety in relation to medicinal cannabis usage (*n* = 23) was more common than framings of medicinal cannabis as a more appropriate therapeutic option than some registered pharmaceutical products, such as opioids (*n* = 7).

There was not much difference between numbers of articles that framed positive research about cannabis (*n* = 19) or those that focused on how evidence about it is poor or lacking (*n* = 24). In contrast, there were only two articles emphasising negative research findings about cannabis. Framings of the challenges for research in the cannabis field occurred in 15 articles.

Not one article suggested that medicinal cannabis should not be legalised; in contrast, 19 articles acknowledged the need for legalisation. Any articles referring to regulation of cannabis for medical use framed regulation as inadequate; no articles shed a positive light on current medicinal cannabis regulation in Australia.

The issue of doctor-patient education was included in the framing codes. Articles framing the need for doctors to be educated about medicinal cannabis occurred in 11 articles, with four articles framing the need for patient education. Scepticism towards traditional knowledge about cannabis use occurred in two articles and, in contrast, one article framed traditional knowledge as positive.

In contrast to the framings about legitimacy mentioned earlier, two articles carried a frame that positioned cannabis as an illegitimate therapeutic option. These were news stories focusing on the use of cannabinoids for chronic cancer pain or inhibiting tumour growth, and mental illness.

## Discussion

Overall, the findings suggest medical publications in Australia construct medicinal cannabis as a legitimate medicine whose regulation is being driven by the community. Cannabis is complex to prescribe and access, does not have a strong evidence base to support its use, and also carries safety concerns. At the same time, the outlook on cannabis research data is largely positive. Findings for primary sources indicate a prioritisation of peer-reviewed journals or government reports, voices from medical associations or foundations, as well as government and university researchers. In the following section we discuss the most prominent frames and sources, those that were less prominent, and intonation.

### Article intonation

Cannabis is frequently framed in positive terms in medical publications in Australia (Fig. 3). These articles framed cannabis as a legitimate medicine that should be legalised and that carried promising research findings. Positive articles also framed the community push for medicinal cannabis as well as the complexities of doctor prescribing and patient access. It is worth noting that negative articles did not appear until 2015, the year before medicinal cannabis was legalised in Australia. The heightened period of negatively toned articles occurred across the period when access was being streamlined across most of the country, with the introduction of the special access scheme and authorised prescriber access. Intonation is further considered in the following sections exploring the framing results.

### Cannabis as legitimate (n = 28)

The articles positively characterise cannabis as a legitimate therapeutic option in the Australian healthcare landscape. In contrast, only two articles during the whole period framed cannabis as an illegitimate substance or approach to therapeutic care. Reports with legitimacy framings start in 2000 with a Medical Journal of Australia ‘Viewpoint’ article about cannabinoids and the endocannabinoid system, which had been discovered 8 years earlier. Legitimacy framings occur in at least three articles a year between 2013 and 2018 and are most predominant in 2014 and 2019 with a total of five articles in those years. These framings occurred most consistently in Australian Doctor (*n* = 17), Medical Journal of Australia (*n* = 5), Medical Observer (*n* = 3), and Australian Medicine (*n* = 3). The items were predominantly news stories (*n* = 20), followed by editorials (*n* = 3), original research or reviews (*n* = 2), one book review in the MJA, and one 2018 case study in Australian Doctor by the country’s first registered cannabis prescribing physician, Dr Vicki Kotsirilos. Reports framing cannabis as a legitimate therapeutic option were predominantly positive in tone (*n* = 21), with fewer (*n* = 6) carrying a mixed tone, and one neutral article.

Professionally speaking, legitimation has been a historically significant process for doctors in Australia and is integral to their social capital reinforcing their elite professional status (Willis 1989). Indeed, this is not just relevant to their professional dominance, but necessarily relates also to the medical objects that doctors integrate into their medical repertoire. As discussed earlier, professional medical publications assist to reinforce the legitimacy of the doctor at the helm of primary healthcare practice in Australia. If doctors are permitted via the legal system to prescribe medicinal cannabis to those patients with specified conditions, then it makes sense that doctors’ publications contribute to legitimising discourse about cannabis through news reports, where it can become integrated into the doctors’ repertoire of medicines and medical objects. The frequency of legitimisation framings in these doctors’ publications is consistent with the findings from the Karanges et al. (2018) study, which found that many GPs regarded cannabis as a legitimate medicine and supported its availability as a prescribed medicine for certain conditions—a medicine that carried potential therapeutic benefit for patients.

The following excerpts offer a glimpse into the legitimisation framings, drawing on notions of scientific evidence and safety, carrying a strong rhetoric of pathos:

A civilised and compassionate country that supports evidence-based medicine and policy should acknowledge that medicinal cannabis is acceptably effective and safe, and probably also cost-effective, especially when the costs of resource use and improvement to the lives and functionality of patients and carers are considered. (MJA, 16 December 2013)

Victorian Health Minister Jill Hennessy said children with severe epilepsy would be the first group to have access to the drug, beginning next year. “We’re starting with these children with severe epilepsy, whose lives have been shown to improve so significantly, because we know these children often don’t make it [into] adulthood,” Ms Hennessy told the ABC. “We want to improve their quality of life.” (AM, 19 April 2016)

This next quotation, from Australian Doctor (2019), invokes the history of penicillin as a ‘natural medicine’ that was accepted by the medical profession as a legitimate medicine some 70 years earlier:

“About 70 years ago another natural medicine came into the medical arena,” writes Professor Nutt, of the Imperial College in London. “This was welcomed enthusiastically by UK doctors, even though there had been no placebo-controlled trials of its efficacy because it was seen to fulfil a major clinical need. “That drug was penicillin. If today’s medical profession could embrace cannabis in the same way as it did penicillin, then the true value of this plant medicine should rapidly be realised.” (Australian Doctor Pharmacy News 7 May 2019).

Legitimacy stories were most commonly associated with framings about the complexity of prescribing, concern about safety issues, the need for legalisation, alternative access issues, positive research findings, and the framing that cannabis is relatively safe. The validity of cannabis as a medicine is also reinforced by the high rate of references to specific conditions: in particular, chronic pain, epilepsy, cancer, and nausea and chemotherapy.

It is rather novel to see a medicine like cannabis be embraced into the mainstream healthcare system in publications that are typically more circumspect towards botanical medicines. This finding contrasts with an earlier study by Lewis (2011a, 2011b) on risk factors in herbal medicine, where a high proportion of doctors found there to be a substantially high rate of reference to risks of plant medicines. Articles typically refer to medicinal cannabis in the context of cannabinoids (notably CBD and THC) as the active constituents, rather than the entourage effect of cannabis sativa as a whole plant (Caldicott et al. 2018). In other words, cannabis is being constructed as a pharmaceutical medicine, rather than a plant medicine. This framing of cannabis as a pharmaceutical is distinct from ‘natural’ or ‘plant’ medicine.

### Regulation being driven by community (n = 28)

Unlike many other medicines, cannabis has bypassed more orthodox methods of medicalisation, in terms of how its legalisation has evolved through an unusually bottom-up trend of patients and advocates seeking legitimisation of access to therapeutic use as opposed to a top-down trend where medical experts legitimise the practice (Bone et al. 2018; Fitzcharles and Eisenberg 2018; Bostwick 2012; Martin and Bonomo 2016). Community-driven regulation was a frequent frame across the study period, acknowledging the role of grassroots activism in the legislation process and recognising medicinal cannabis advocacy as part of a social movement. This has been a significant feature of cannabis advocacy around the world, which has seen legalisation of cannabis as an outcome of the work of community-based activists ranging from patients and carers to community dispensaries, occasionally supported by local governments (Penn 2014; Blickman 2014; Frankhauser 2008). This frame did not appear until 2013, a couple of years before new legislation about medical access, reaching its peak in reports in 2017 and 2018, and demonstrating a decline in reports in 2019, once legislation was well established. The tone of these stories is typically positive or mixed:

A group of Queensland mums are seeking to put medical cannabis on the state election agenda, claiming it could “save” their children.

-Hoffman, Tessa ‘Mums lobby for legal cannabis for kids’ Australian Doctor 2015

In the aftermath of this flurry of activity there has been widespread confusion and scepticism among doctors. Many question whether the cart has been put before the horse; whether legislative change has been driven by the passionate campaigns of patients and advocacy groups rather than evidence-based medicine.

-Dunn, Emily ‘Your guide to the clamour for cannabis’ Australian Doctor 2017

Medicinal cannabis certainly has had a very political and community driven introduction in this country.

-AMA Vice President Dr Toney Bartone, ‘Medicinal cannabis – still a lot of misinformation’ Australian Medicine 2017

Like complementary medicine advocates, medicinal cannabis activists have worked to challenge existing policies on cannabis and advocate and even conduct their own research to understand its efficacy, benefits, viability, and social value. This is a strong feature of health social movements (Brown and Zavestoski 2004) and regular reference to the ‘driven by the community’ framing in these articles conveys an awareness—which may not necessarily be manifestly articulated—that medicinal cannabis is a medicine being strongly advocated by citizens such as ‘Queensland mums’ (Hoffman 2015), or ‘patients and advocacy groups’ (Dunn 2017).

### Complexity of prescribing and patient access (n = 25)

These articles make salient the point that the process by which medicinal cannabis is prescribed is exceedingly complex for doctors and their patients. For example:

…there are several hurdles to be jumped before the script can be actioned. First, doctors will first need to be approved by the government as registered prescribers of cannabis-based medicine. Then, for each patient’s prescription, they will need to apply for approval to the TGA and NSW Health, providing clinical evidence to support their application. Doctors are also expected to have tried other medical and nonmedical interventions before resorting to a cannabis-based product.…AMA NSW spokesman Clinical Associate Professor Saxon Smith…says that while the rules come into effect on Monday, the implementation process will be lengthy and it could be months before the first script is approved…

-Hoffman, Tessa ‘Green light for unapproved cannabis scripts’ Australian Doctor 2016

For doctors trying to support patients, navigating medicinal cannabis prescribing pathways can be torturous. It is still up to individual practitioners to reconcile the clinical evidence, come to a view about its therapeutic appropriateness and complete the appropriate processes for accessing medicinal cannabis products.

-Gill, Kate and Brell, Ruanne ‘How to navigate the logistical labyrinth that is medicinal cannabis’ Medical Observer 2018

The high rate of references to ‘access’ (*n* = 190) and derivatives of the word ‘prescribe’ (*n* = 303) generated by word mapping correlate with the frequency of framings about the complexity of prescribing and patient access. Practitioners can register with the TGA for special prescriber access in order to be able to prescribe for large numbers of patients without seeking TGA approval for each individual patient. Alternatively, for the Special Access Schemes (SAS) A and B,^3^ they make a case to the regulator for each individual patient for whom they wish to prescribe medicinal cannabis. This submission process has two effects. The first is that doctors are resistant to becoming prescribers because of the complexity and difficulty of the process and the labour required in order to either become registered or to make submissions for individual cases. Doctors are forced into a position of making a crude, self-cost-benefit analysis as to the worth of undertaking this process. The second element is that owing to the difficulties of following legitimate prescription routes, a natural consequence of this will be for patients to seek remedies through self-prescription and self-medication. This creates a double regulatory bind. On the one hand, we have the resistance by practitioners because of the complexity; on the other, we have the illegal route taken by self-prescribing patients because of the challenges of trying to obtain prescriptions from a doctor. Even in countries where legalisation of cannabis is total, its therapeutic uses are held back by recognisable obstacles, such as lack of education (St Pierre et al. 2020), stigmatisation (St Pierre et al. 2020; Balneaves et al. 2018), and regulatory hurdles (Abuhasira et al. 2018)

### Safety concerns (n = 23)

Concern about safety issues for medicinal cannabis use was a frame in the study with word mapping showing the words ‘risk’ or ‘risks’ occurring 152 times across articles, ‘safety’ or ‘safe’ were used 134 times, and ‘harm’ or ‘harms’ 46 times. It is important to note that the most concentrated periods for stories framing safety concerns about cannabis were in 2019 (*n* = 7) and 2018 (*n* = 4), with three items carrying such framings in 2016 and 2017. This indicates that the focus on cannabis risk is a more recent phenomenon articulated across these medical publications, and understandably coincides with the period following legislation, after which increasingly aware patients were requesting legal access to cannabis and doctors were starting to register and prescribe it.

In an article from 2016, ANU physician and researcher, Dr David Caldicott, addresses the anxieties of doctors in this field (Woodhead 2016):

“The message for clinicians is that they shouldn’t be afraid. There are already very well-developed [medical cannabis] markets out there, so we don’t have to reinvent the wheel. It’s very hard to argue that you are going to harm anybody with a regimen that will be very tightly regulated. And clinicians should not be expected to prescribe something with which they are not comfortable or prescribe something prior to them having the opportunity to learn everything they want to know about it.”

This can be contrasted with another 2016 article from Medical Observer quoting an addiction specialist based at the Royal Adelaide Hospital, who articulated a strong level of safety concern for potential harm on brain development in children and young people:

Medicinal cannabis trials in children and teens should not go ahead unless there is evidence they won’t cause long-term harm to the developing brain, an addiction medicine specialist says.

-Worsley, Rachel ‘Addiction expert slams medicinal cannabis for kids’ Medical Observer 2016

Doctors are understandably highly attuned to matters of risk and their awareness of it constantly underpins much of their practice. Accompanied by efficacy and quality, safety is a primary category used to assess the viability of new medicinal substances at the regulatory level as being integral to evidence-based medicine, not to mention a fundamental principle of primary health care. As a new medicine—and a plant-based one at that—it is perhaps surprising that framings about safety concerns were not more prominent across these publications. This finding can be contrasted with a study of MJA articles about herbal medicine across a 42-year period (Lewis 2011a, 2011b), which found that the majority of MJA items in the study referred to herbal medicine risk. It is unclear whether doctors are feeling completely reassured by laboratory and clinical trial research into medicinal cannabis. The dearth of articles addressing traditional usage or acknowledging validity of traditional usage suggests that this may not be a highly valued attribute in doctors’ publications.

Overall, the safety concern framings did not negate the use of medicinal cannabis, and arguably functioned to demonstrate the legitimacy of cannabis as a pharmacological and pharmaceuticalised substance, as reflected here:

“We need to have proper trials and regulate it as a medication just like any other medication…It’s not about trying to deny access to the drug, but we also want to make sure that we don’t do any harm. We want to make sure that people are actually getting the drug for the right reasons, and that it’s actually going to benefit them in the future.”

-Rollins, Adrian ‘Cannabis meds? Follow the evidence, says AMA’ Australian Medicine 2015

This point about pharmaceuticalisation is reinforced by the word mapping, which revealed very substantial distinctions between the comparatively low frequency of the word “plant” (*n* = 28) and more pharmaceutically-oriented terms, such as “cannabidiol” (*n* = 154) or “CBD” (*n* = 142), and “THC” (*n* = 106) which as discussed earlier refer to particular isolated active constituents (cannabinoids) contained in the whole plant. A purely pharmaceuticalised orientation towards medicinal cannabis discourse is, however, not so straightforward. Across the period, images of the whole plant and raw plant materials were far more common than photographs of the manufactured and bottled product or of clinical research or laboratory images. This could relate to the availability, access, and cost of using particular images for publishers, and undoubtedly the aesthetic appeal of the bright, green cannabis foliage. At the same time, it highlights a tension between cannabis as a pharmaceutical substance or a ‘drug’ and cannabis as a plant.

With the exception of articles in the MJA, cannabis safety concerns were often discussed generally, rather than specifically, expressing the safety concerns about cannabis as an unfamiliar and unknown entity, rather than something proven to be dangerous. When specifics were mentioned it related to long-term usage, dosage, and side-effects. The concern about safety based on lack of familiarity, rather than confirmed risks of cannabis as a medicine, is echoed in research of doctors’ attitudes towards unconventional therapies by Newell and Sanson-Fisher (2000) and Lin et al. (2005) and a more recent study that highlighted concerns from gastroenterologists in Australia (Benson et al. 2020).

A previous study of representations of herbal medicines in the MJA across a 42-year period by Lewis (2011a, 2011b) indicated that the most frequent references to risks about herbal medicines were associated with adverse events and toxicity in particular, as well as a lower frequency of mentions of drug interactions, and dosage. Word text searches within NVivo for this study indicated a higher reference to dosage (*n* = 23), followed by adverse events (*n* = 15) and toxicity (*n* = 9), and drug interactions (*n* = 4).

Importantly, the framing of concerns about safety did not function to delegitimise the value of cannabis as a medicine. Whilst concerns are articulated about safety in 20% of reports, cannabis appears to be a trusted medical object in these doctors’ publications.

### Framings of research evidence (poor evidence n = 24 and positive findings n = 19)

Framings of research evidence in these articles demonstrate a pattern of acceptance of cannabis that is correlated with the level of evidence available for the particular condition it is treating. This is consistent with the findings from Karanges et al. (2018). The framings on positive or promising evidence, as well as the distinct concern about the lack of evidence, are an acknowledgement that evidence to support medicinal cannabis usage is a necessary and desirable outcome. Rare are the framings of negative research findings that suggest cannabis is not safe, efficacious or effective, or an invalid therapeutic option. Given that cannabis is a plant-based product, which in the regulatory context may consist of a standardised extract of CBD or THC, a synthetic version of either of the two, or a whole plant extract, the apparent openness in these publications to the potentialities of cannabis might be contrasted with the response to a product like *Hypericum performatum* (St John’s Wort), which has a solid evidence base for use in people with mild to moderate depression (Linde et al. 2008). Despite this evidence, St John’s Wort is not supported in primary care medicine in Australia, nor is it a registered medicine. An important question here might be: why is medicinal cannabis regarded as more valid and viable than another plant that has sound evidence of efficacy to support its usage, like St John’s Wort? What makes cannabis so distinct from another plant medicine like St John’s Wort? This is a question for further research but it is not merely a matter of evidence, but also sociocultural and political matters, not to mention economics. The matter of standardisation is also relevant, given the pharmacological complexity of whole plant medicines in comparison to the process of isolating active constituents and creating a standardised extract that has eliminated the wide variabilities of the whole plant. Arguably, the representation of medicinal cannabis as a controllable, ‘pharmaceuticalisable’ product is an important part of the discourse on safety and evidence. Framings that directly addressed standardisation, however, were surprisingly few (*n* = 5). We shall now look at some of the less common frames.

### Less common frames

The scale of the opioid crisis and accompanying news coverage that depicts the risks and harms caused by the over-prescription of opioids and the influence of pharmaceutical companies in promulgating this culture of over-prescription has been huge (Stoicea et al. 2019). When we searched using the term ‘opioid crisis’ across Australian Doctor issues, for example, we found 57 articles. Thus, we had anticipated more articles addressing cannabis as an alternative option for pain relief in these publications. While there were seven such reports, all of which referred positively to the potentialities of cannabis as an alternative to opioids for pain relief, it is unclear why this framing was less common. It may be due to caution given the lack of robust safety and efficacy data about cannabis in treating pain (Stockings et al. 2018; Moore et al. 2021), although the references to cannabis and chronic pain in this study are substantial.^4^ Moore et al. (2021:S76), however, argue that pain specialists should be included more in such research, for example:

It is telling that a U.S. National Academies of Sciences, Engineering and Medicine report on therapeutic effects of cannabis and cannabinoids, and a later update,^5^ concluded that there is “substantial” evidence that cannabis is an effective treatment for chronic pain in adults. The committee included experts in substance abuse, cardiovascular health, epidemiology, immunology, pharmacology, pulmonary health, neurodevelopment, oncology, pediatrics, public health, and systematic review methodology, but not pain.

Framings that convey commercialisation concerns were surprisingly rare in this study. This is noteworthy, given the scrutiny that has arisen from doctors’ groups in Australia targeting complementary medicines and botanical and nutritional supplements, in particular (Lewis 2019, 2020). The narrative of cannabis as a legitimate and new medicine, laden with not just therapeutic, but commercial possibilities, seems to have evaded such scrutiny and critique, with the exception of one Australian Doctor article, which commented:

But National Cannabinoid Clinics’ financial links with Tilray create unique ethical issues for GPs to navigate,^6^ says Associate Professor Vicki Kotsirilos, a former chair of the RACGP integrative medicine group. (Ausdoc, 10 April 2019, by Geir O’Rourke)

It is possible that doctors are still grappling with the efficacy and benefits of cannabis, along with its risks and all its access and prescribing complexities, before turning their attention to matters of commercialisation, conflicting interests, and ethics. It also may be that for a profession highly familiar with a pharmaceuticalised approach to healthcare, the medicinal cannabis model offers an easy logic, whereby it can be embraced as a medicalised, commercialised, pharmaceutical substance rather than anything resembling herbal medicine.

## Conclusion

Medicinal cannabis in Australia is neither fully endorsed nor rejected as a therapy. This probably stems from two lines of thought. The first is focussed on the perceived and actual illegality of cannabis in many places. It is confounded also by its notorious method of administration; smoking. Medicinal cannabis, however, depends on neither of these aspects: it is legal and it is delivered in non-harming ways. Still, many politicians are averse to endorsing cannabis as legal or medical. Its progress into the mainstream remains fraught. The second line of thought is to do with the amount of research on the therapeutic medical benefits of cannabis. Governments are beginning to invest more in research as they are with other previously “untouchable” drugs, e.g. ketamine, psilocybin, and LSD.^7^ The result is that clear evidence is not available in all spheres of medicinal cannabis, and much of its presumed benefits rely on the testimonies of patients and others. Nevertheless, with increased research the evidence is becoming stronger. Both lines of thought have to contend with one significant fact: the illegal market remains the dominant mode of cannabis production and consumption. The piecemeal reform of the medicinal and recreational cannabis markets will continuously face these pressures until the market is reformed. Commercial growers and producers of cannabis products are intensifying their efforts to transform regulation and open up the market thereby simplifying procurement and access. This is all part of a delicate balance between market, medicine and the state, yet to find its resolution.

Our research shows the dominant framings about medicinal cannabis in doctors’ professional publications position cannabis as a valid medical substance, acknowledging the significant role played by the community in its legalisation, hence its role as a new medicine to which doctors have exclusivity. Despite its legitimacy, it is also largely acknowledged as having a weak evidence base, although framings of positive research findings outweigh negative ones. Cannabis is also framed for its potential risk, though as a more general concern based on what is not known about it, rather than specific articulated risks. Chronic pain or pain were the conditions most frequently mentioned in articles about cannabis, followed by epilepsy, cancer or cancer pain, and nausea and chemotherapy. It is novel to see a medicine like cannabis be embraced into the mainstream healthcare system in publications that are typically more sceptical towards botanical medicines especially given the articulated understanding of the underlying pharmacological complexity. A limitation of this study is its focus on general practitioner publications, whereas the discourse across specialist publications may yield quite different findings.

Our research provides a springboard for future exploration of the phenomenon of cannabis’ mainstreaming through sociological and communication approaches, as its mediatisation in publications like professional medical magazines and journals plays such an integral role in its validation and legitimation. While this research is limited to content analysis, audience effects research will add to our understanding of medicinal cannabis mediations, offering valuable insights about how doctors receive and respond to such mediations. We further believe this research should be expanded to other drugs as mentioned above, such as ketamine and psilocybin. The distinctions between licit and illicit will have to be renegotiated as new therapies enter the mainstream and how people respond to this will require careful management and communication.

As a relative newcomer to the discourse in Australian medical publications, medicinal cannabis is being constructed as a valid medicine with potential for addressing a range of conditions despite the lack of evidence and a medicine which (like many registered prescription medicines) is not risk-free. These publications also acknowledge the role of the community in the legalisation of medicinal cannabis, which is largely framed as having a legitimate place in mainstream Australian healthcare. We conclude that developments in new therapies will only be successful if they are matched with concomitant progress in disseminating news and communication to both practitioners and patients.

## Data Availability

We are happy to make available the media dataset from this study and the coding information and codesheet.

## Abbreviations

ACT: Australian Capital Territory
AFP: *Australian Family Physician*
AM: *Australian Medicine*
AMA: Australian Medical Association
ANU: Australian National University
Ausdoc: ausdoc.com.au
CAMS-18: Cannabis as Medicine Survey
CBD: Cannabidiol
GP: General practitioner
MJA: *The Medical Journal of Australia*
NCSL: National Conference of State Legislatures (US)
NSW: New South Wales
ODC: Office of Drug Control
RACGP: Royal Australian College of General Practitioners
TGA: Therapeutic Goods Administration
THC: Tetrahydrocannabinol

## Footnotes

1 Australian Family Physician (AFP) was the peer-reviewed publication of the Royal Australian College of General Practitioners and was superseded by Australian Journal of General Practice from 2018, https://www.racgp.org.au/afp/.

2 Intonation of both headline and article were coded based on whether the *overall framing and tone towards the use of medicinal cannabis as a therapeutic option* was positive, negative, neutral, or mixed.

3 https://www.tga.gov.au/book-page/information-health-practitioners

4 It is worth noting that one of these articles was a report sponsored by a medical research and development company designing a range of cannabinoid-based products (Australian Doctor Group 2018).

5 Committee on the Health Effects of Marijuana 2017; Abrams 2018.

6 Tilray is identified in the article as “a Canadian company that sells cannabidiol (CBD) and tetrahydrocannabinol (THC) extracts to approved Australian patients for up to $420 per 25 mL vial”

7 For example, see Ketamine for adult depression study, https://www.australianclinicaltrials.gov.au/anzctr/trial/ACTRN12616001096448; and Psilocybin-assisted psychotherapy for the treatment of depression and anxiety associated with life-threatening illness, https://www.australianclinicaltrials.gov.au/anzctr/trial/ACTRN12619001225101.
