# Correction to: The transition of cannabis into the mainstream of Australian healthcare: framings in professional medical publications

**DOI:** 10.1186/s42238-021-00110-z

**Published:** 2021-12-15

**Authors:** Monique Lewis, John Flood

**Affiliations:** Grifth University, Grifth, Queensland Australia

**Correction to: J Cannabis Res 3, 48 (2021)**

**https://doi.org/10.1186/s42238-021-00105-w**

Following the publication of the original article (Lewis & Flood, 2021), the author reported that Figures 3 and 4 in the HTML version were incorrect.

The original article has been updated.
